# Anesthetic Challenges and Perioperative Factors Affecting Delayed Graft Function in Robotic-Assisted Kidney Transplant: A Review of a Single-Center Experience of 100 Cases

**DOI:** 10.7759/cureus.28957

**Published:** 2022-09-08

**Authors:** Nisha Rajmohan, Shilpa Omkarappa, Sangeeth P Srinivasan, Suresh G Nair, Rajesh Rajgopal, Nidhin Eldo

**Affiliations:** 1 Department of Anesthesiology and Critical Care, Aster Medcity, Kochi, IND

**Keywords:** delayed graft function, pneumoperitoneum, trendelenburg position, kidney transplantation, robot assisted renal transplant

## Abstract

Background and objective

The advent of robot-assisted kidney transplant (RAKT) has ushered in a new set of challenges. In this single-center retrospective observational study, we aimed to highlight the anesthetic challenges and analyze perioperative parameters to identify the risk factors for delayed graft function (DGF) in RAKT.

Methods

A descriptive analysis of perioperative factors of the first 100 cases of RAKT at our center was performed. Data were retrieved from the hospital's electronic medical records (EMR) of donors and adult patients who underwent RAKT between July 2015 and December 2020. The data analyzed included demographics, preoperative optimization, intraoperative and postoperative management, and complications. DGF was defined as a requirement of dialysis within one week of transplant. The Fisher’s exact test, independent sample t-test, and the Mann-Whitney test were used to analyze data.

Results

Among a total of 193 renal transplants performed during the study period, 100 patients underwent RAKT, which included 27 females and 73 males. Of these, 91 were live while the remaining involved deceased-donor transplants. Pneumoperitoneum and steep Trendelenburg position required various “anesthetic maneuvers” to maintain hemodynamics and respiratory parameters. Optimal fluid management, with frusemide and mannitol, ensured good urine output (UOP) (93%). Post-reperfusion, the release of pneumoperitoneum, maintenance of adequate perfusion pressures, immunosuppression, and regional hypothermia helped in ensuring adequate graft function (93%). The incidence of DGF in our series was 7% and the mortality rate was 3%. Recipient age (p=0.045), dyslipidemia (p=0.021), and diabetes mellitus (p=0.023) were identified as significant risk factors for DGF.

Conclusion

Advanced recipient age, diabetes, and dyslipidemia were factors significantly associated with DGF in RAKT in our series of 100 cases. However, the duration of the steep Trendelenburg position, docking of robot/pneumoperitoneum (console time), fluid management, warm and cold ischemia times, rewarming time, and type of graft did not influence DGF. Awareness of the systemic involvement in RAKT, proper preoperative optimization, and knowledge of potential problems are essential for the efficient anesthetic management of RAKT.

## Introduction

The emergence of surgical robotic systems has opened up new dimensions in transplant surgery [1]. Anesthetic challenges that are significant during renal transplants get compounded during robotic-assisted kidney transplants (RAKT) [2]. Traditionally, fluid regimen, graft characteristics, anesthetic technique, comorbidities, and cold ischemia time (CIT) were some of the factors that predisposed to delayed graft function (DGF) in open renal transplants [3]. Longer duration of surgery, increased CIT, rewarming time, absence of a proper fluid regimen, and the effect of pneumoperitoneum on graft can increase the risk of DGF in RAKT. There is scarce literature on anesthetic management in RAKT, and the same is true for the data on the incidence of and predisposing factors for DGF. In this single-center retrospective cohort study, we highlight the perioperative challenges, rate of incidence, and perioperative risk factors for DGF in RAKT.

## Materials and methods

This was a single-center retrospective cohort study of the first 100 cases of RAKT treated at our tertiary care center. The electronic medical records (EMR) in the operating room and intensive care unit (ICU) pertaining to patients who underwent RAKT from July 2015 to December 2020 at our center were evaluated after obtaining institutional approval (AM/RC/90-2019). The study was conducted in accordance with the principles of the Declaration of Helsinki. The aim of the study was to identify the anesthetic challenges and to find out the incidence and perioperative risk factors of DGF. All adult patients who underwent RAKT were included in the study. The open renal transplant patients and the pediatric recipients were excluded. The data analyzed were as follows: demographic profiles; indication for transplantation; comorbidities; duration of dialysis; preoperative hemoglobin, creatinine, and potassium levels; duration and technique of anesthesia and surgery; console time; volume and type of intravenous fluids; surgical findings; warm ischemia time (WIT); rewarming time; CIT; intraoperative urine output (UOP); immunosuppressant therapy; antibiotics; timing of extubation; perioperative complications; the need for postoperative dialysis; serum creatinine levels on postoperative days (POD) three, five, and seven, and at one month and one year; and duration of ICU and hospital stay.

Kidney failure immediately after transplantation, requiring dialysis in the first week post-transplant, was defined as DGF [3]. WIT was defined as the time from clamping of the renal artery to the start of perfusion. Secondary warm ischemia/rewarming time was defined as the time from graft insertion into the body to reperfusion. CIT was defined as the time from the start of cold perfusion to graft insertion into the body. The console time was defined as the time from the docking of the robot to undocking, which also corresponded to the duration of pneumoperitoneum. The incidence of DGF was considered the primary endpoint. Results were compiled into a series of Excel sheets to be analyzed. We compared the characteristics and evolution of patients who presented with DGF versus recipients free of this complication.

Anesthesia technique

Ondansetron (4 mg), pantoprazole (40 mg), alprazolam (0.25 mg), and antihypertensive drugs were given to the patient on the night before and in the morning of surgery. Immunosuppression protocol included mycophenolate mofetil (35 mg/kg) and tacrolimus (0.1 mg/kg) initiated two days prior to surgery, and basiliximab 20 mg was added in cases of deceased-donor transplant and donors who were not first-degree relatives. All the patients received methylprednisolone (7.5 mg/kg) intraoperatively. All live-donor recipients were dialyzed 24 hours pretransplant and the deceased-donor recipients were dialyzed at the earliest before the transplant. After the dialysis, preoperative investigations, namely, hemoglobin, total count, platelet count, prothrombin time, serum electrolytes, and renal function tests, were performed.

In addition to standard monitoring, an arterial line, central venous triple lumen catheter, and a bispectral index (BIS) monitor were used in all patients. General anesthesia (GA) was induced with midazolam (0.02-0.04 mg/kg), fentanyl (3-4 mcg/kg), propofol (1-2 mg//kg), and atracurium (0.5 mg/kg). Anesthesia was maintained with air, oxygen, isoflurane, atracurium (0.5 mg/kg/hour), and dexmedetomidine infusions (0.2-0.5 mcg/kg/hour). Isoflurane was titrated to maintain BIS between 40 and 60. Patients were mechanically ventilated using pressure control ventilation (PCV), peak pressures limited to less than 35 mmHg, positive end-expiratory pressures (PEEP) of 5 mmHg, and the rate adjusted to maintain normocarbia. The temperature was maintained by fluid warmers, hot-air warmers, and warming blankets. Intermittent pneumatic compression devices were used for deep vein thrombosis (DVT) prophylaxis, and a nasopharyngeal tube was inserted.

A restrictive fluid management strategy (1 ml/kg/hour) of crystalloids was used in all patients till the implantation of neo kidney, followed by a more liberal fluid regimen (1.5-1.75 ml/kg/hour). This resulted in 1.5-2 liters of fluids being administered before reperfusion. Both normal saline and Ringer's lactate were used in these patients. Post-reperfusion, the fluid intake was based on the previous hour's UOP plus 200 ml. A combination of furosemide (100 mg) and 20% mannitol (0.25-0.5 mg/kg) was used before reperfusion.

In addition to intravenous fluids, noradrenaline was used to maintain adequate mean arterial pressure (MAP) after reperfusion. In three (3%) cases, nitroglycerine (NTG) was used to control intraoperative hypertension.

A single surgeon, experienced in the Da Vinci systems (Intuitive Surgicals, Sunnyvale, CA) operated on all patients. Pneumoperitoneum was created by insufflating CO_2_ through the Veress needle. A 30-45-degree Trendelenburg position was executed and the robot was docked. Central venous pressure (CVP) increased by 14 ± 4 mmHg. The intra-abdominal (IAP) pressure was maintained at 15 mmHg. After reperfusion, the pneumoperitoneum was completely released for open ureteric anastomosis.

Extubation was attempted in all except eight patients (8%). Seven patients were not extubated on the table as they were deceased-donor transplants, which were started at odd hours. One patient was a case of anticipated difficult intubation who was extubated once, fully awake in the ICU. Postoperative analgesia was provided by fentanyl infusion (0.5 mcg/kg/hour) and intravenous paracetamol 1 gram thrice daily. The patients were discharged from the hospital once their creatinine levels stabilized.

Statistical analysis

For categorical variables, we presented the data as numbers and percentages. For continuous variables, we presented the mean and standard deviations (SD). In situations where the continuous data were skewed, the median and range were reported. The Fisher’s exact test was used to compare the categorical variables with postoperative complications. The independent sample t-test was used to compare the continuous variables with postoperative dialysis status. The Mann-Whitney test was used to compare the duration of dialysis and one-month postoperative creatinine with postoperative dialysis status. A p-value <0.05 was considered statistically significant. We used Bonferroni correction for presenting p-values related to multiple comparisons. Statistical analyses were performed using SPSS version 20.0 for Windows (IBM Corp., Armonk, NY).

## Results

Of the 193 renal transplants performed in total, 100 were RAKT. There were 27 females and 73 males. Ninety-one were live while the remaining involved deceased-donor transplants. All except deceased donors underwent laparoscopic nephrectomies. A custodial solution was used for perfusion. The most common indication for transplant was chronic glomerulonephritis followed by IgA nephropathy. The demographic details and comorbidities of the cohort are presented in Table 1.

**Table 1:** Demographic variables and comorbidities *Gout and systemic lupous erythematosus CVA: cerebrovascular disease; LV: left ventricular; SD: standard deviation

Perioperative recipient factors	Requirement of postoperative dialysis		P-value
	No, n (%)	Yes, n (%)	
Gender			0.671
Female	26 (28.0)	1 (14.3)	
Male	67 (72.0)	6 (85.7)	
Type of graft			0.120
Live	86 (92.5)	5 (71.4)	
Deceased	7 (7.5)	2 (28.6)	
Hypertension			1.000
No	5 (5.4)		
Yes	88 (94.6)	7 (100.0)	
Diabetes mellitus			0.023
No	78 (83.9)	3 (42.9)	
Yes	15 (16.1)	4 (57.1)	
Dyslipidemia			0.021
No	86 (92.5)	4 (57.1)	
Yes	7 (7.5)	3 (42.9)	
Hypothyroidism			1.000
No	82 (88.2)	7 (100.0)	
Yes	11 (11.8)		
Coronary artery disease			0.093
No	79 (84.9)	4 (57.1)	
Yes	14 (15.1)	3 (42.9)	
Valvular heart disease			
No	89 (97.8)	7 (100.0)	1.000
Yes	2 (2.2)		
LV dysfunction			0.096
No	87 (93.5)	5 (71.4)	
Yes	6 (6.5)	2 (28.6)	
Cardiomyopathies			1.000
No	89 (97.8)	7 (100.0)	
Yes	2 (2.2)		
Others*			0.664
No	64 (68.8)	5 (83.3)	
Yes	29 (31.2)	1 (16.7)	
CVA		7 (100.0)	1.000
No	90 (96.8)		
Yes	3 (3.2)		
Use of basiliximab			0.112
No	58 (62.4%)	2 (28.6%)	
Yes	35 (37.6%)	5 (71.4)	

Of note, 94% of patients were on a thrice-weekly dialysis regimen with a quarter of them being on dialysis for more than one year; 47% of patients were started on dialysis within six months. The intraoperative and postoperative parameters are detailed in Tables 2-3. Left ventricular hypertrophy (LVH) was the most common finding on echocardiograms (92%); 8% of patients had normal echocardiogram findings.

**Table 2:** Perioperative parameters and delayed graft function BMI: body mass index; CIT: cold ischemia time; ICU: intensive care unit; POD: postoperative day; SD: standard deviation

Recipient factors	Delayed graft function						P-value
	No			Yes			
	N	Mean	SD	N	Mean	SD	
Age (years)	93	41.3	11.7	7	50.4	6.6	0.045
Preoperative hemoglobin (gm/dl)	93	10.2	1.7	7	10.6	1.3	0.581
Preoperative creatinine (mg/dl)	93	8.9	3.0	7	10.2	5.1	0.297
Preoperative potassium (meq/l)	93	4.7	0.7	7	4.9	1.0	0.511
Total fluids (ml)	92	2680.3	801.4	7	2571.4	672.6	0.727
Ringer's lactate (ml)	59	983.9	557.7	4	1375.0	750.0	0.188
Normal saline (ml)	92	2057.1	1056.5	7	1785.7	906.3	0.511
Intraoperative urine (ml)	90	909.4	415.2	6	691.7	521.9	0.224
Duration of anesthesia (minutes)	93	359.5	48.8	7	368.6	87.8	0.797
Duration of surgery (minutes)	93	258.1	48.5	7	268.0	81.8	0.763
Creatinine at POD 3 (mg/dl)	93	1.4	0.7	6	4.6	1.9	0.011
Creatinine at POD 5 (mg/dl)	93	1.3	0.6	6	4.6	1.7	0.006
Creatinine at POD 7 (mg/dl)	92	1.4	0.5	6	4.4	2.2	0.019
Creatinine at 1 year (mg/dl)	85	1.3	0.5	4	1.6	0.3	0.209
BMI	93	26.4	17.4	7	27.4	4.7	0.876
Console time (minutes)	92	164.8	38.4	7	189.9	69.5	0.381
CIT (minutes)	92	65.8	46.3	7	77.3	43.5	0.525
WIT (minutes)	92	4.3	1.5	7	5.1	1.5	0.135
Rewarming time	71	41.6	9.8	7	45.9	14.5	0.295
Duration of ICU stay (days)	93	7.4	1.7	7	11.7	2.7	<0.001
Duration of hospital stay (days)	93	9.7	2.1	7	12.6	2.8	0.001

**Table 3:** Recipient parameters and requirement of postoperative dialysis

Recipient factors	Postoperative dialysis						P-value
	No			Yes			
	N	Median	Range	N	Median	Range	
Duration of dialysis (months)	91	7.0	1-48	7	12.0	2-24	0.487
Creatinine at one month post surgery (mg/dl)	91	1.25	0.69-3.83	6	2.07	1.53-6.90	0.001

Conversion to open occurred in three (3%) cases because of the presence of an arterial thrombus, acute venous kink, and decreased perfusion in the lower renal pole. One patient required a dual kidney transplant from a deceased donor and two patients had vaginal insertion of the graft. Double renal artery implantation had to be performed in 12% of patients. Three (3%) patients had hyperkalemia before reperfusion, which was aggressively managed. Eight patients were extubated in the ICU.

Gram-negative sepsis, polyuria leading to hypernatremia, impaired level of consciousness and aspiration pneumonia, and ureteric leak after stent removal were the postoperative complications we encountered. After extubation, none of the patients had airway issues although most had facial puffiness. Seven (7%) patients had DGF, necessitating two to three cycles of hemodialysis. Three patients had graft rejection, of which two were successfully managed. There were no wound infections, lymphocele, or prolonged ileus. The mean blood loss was 88 ± 51 ml.

One patient was re-explored for postoperative bleeding, four hours after surgery. There were three (3%) deaths in the entire cohort. Dengue hemorrhagic fever, DGF with bacterial sepsis, and antibody-mediated rejection with sepsis were the causes of mortality. The analysis of perioperative factors revealed that there was a significant association of DGF with recipient age (p=0.045), dyslipidemia (p=0.021), and diabetes mellitus (0.023) (Tables 2, 3).

The creatinine value was statistically higher in patients who had DGF on day three (p=0.011), day five (p=0.006), day seven (p=0.019), and at one month (p=0.001), but there was no difference by the end of one year. We did not get serum creatinine from seven patients at one year as they were lost to follow-up. We found that the duration of ICU stay and hospital stay were higher in the DGF group (p<0.001). We could not find an association between donor factors, namely, age, gender, comorbidities, and type of graft (Table 4).

**Table 4:** Donor factors and delayed graft function SD: standard deviation

Donor factors	Delayed graft function requiring hemodialysis		P-value
	No	Yes	
Gender, n (%)			0.054
Female	67 (95.7)	3 (4.3)	
Male	26 (83.3)	4 (16.7)	
Age, years, mean ± SD	47.5 ± 11.8	52.1 ± 6.0	0.084
Comorbidity, n (%)			0.198
No	71 (89.9)	7 (10.1)	
Yes	22 (100.0)	-	
Relationship, n (%)			0.076
Cadavers	7 (70.0)	2 (30.0)	
Relation live	61 (95.2)	3 (4.8)	

Logistic regression model for postop dialysis with independent risk factors could not be used since the number of incidences was less corresponding to the number of risk factors associated.

## Discussion

Anesthetizing patients for RAKT poses a great challenge because of the multisystem involvement of end-stage chronic kidney disease (CKD), the effect of pneumoperitoneum and Trendelenburg position on hemodynamics and fluid management, possible deleterious effects of pneumoperitoneum on the neo kidney, and difficulties associated with robotic surgery [2].

LVH, coronary artery disease (CAD), arrhythmias, pulmonary hypertension, diastolic dysfunction, pericardial effusion, uremic cardiomyopathy, anemia, coagulopathy, and electrolyte and acid-base disturbances are risk factors associated with anesthesia for CKD [2]. An early, multidisciplinary preoperative optimization strategy ensured that our patients presented in the best possible condition. Preoperative dialysis at the appropriate time helped prevent heparin- and uremia-related bleeding and hypotension at the induction of anesthesia.

GA in steep Trendelenburg with lithotomy position and pneumoperitoneum can lead to reduced functional residual capacity and lung compliance, promote atelectasis, and generate high airway pressures with reduced minute ventilation, hypoxia, and hypercarbia [2,4]. Our mode of ventilation (pressure control) with adequate minute ventilation helped prevent hypercarbia and hypoxia. Plateau pressures were maintained below 30-32 mmHg and a PEEP of 5 mmHg was ensured.

Carbon dioxide pneumoperitoneum with a steep Trendelenburg position can alter the normal end-tidal carbon dioxide (ETCO_2_)-to-PaCO_2_ relationship (4-6 mmHg). Restoration of normal ETCO_2_-to-PaCO_2_ relationship after surgery may take several hours [2]. Because of the underestimation of PaCO_2_ by ETCO_2_, and the long duration of our surgeries (257 ± 50.74 minutes), we based our ventilation strategy to achieve a normal PaCO_2_. Therefore, an arterial line and arterial blood gas analysis were used in all. Hypercarbia and respiratory acidosis have to be avoided to prevent associated hyperkalemia, raised intracranial pressure (ICP), cardiac depression, and dysrhythmia. Hyperkalemia before reperfusion was seen in only 3% of patients, probably due to the administering of preoperative dialysis and avoidance of hypercarbia.

A study on laparoscopic renal transplants found that pneumoperitoneum caused a significant increase in MAP, which necessitated the initiation of NTG in 65% of patients [2]. An increase in systemic vascular resistance (SVR) induced by the effects of pneumoperitoneum on the aorta and arterial elastance translates into significant increases in MAP [2,5]. In our series, we found that only three patients required treatment with NTG infusions for the management of intraoperative hypertension. The use of dexmedetomidine in our patients could have contributed to the absence of a hypertensive response.

Renal graft function and survival are directly influenced by the transplanted graft perfusion [2]. In our series, we maintained a MAP of 30% above baseline by using norepinephrine and by appropriate fluid management. The appropriate immunosuppression helped prevent graft rejection in 97% of patients.

In our series, even though peri-orbital and facial edema was noticed, we did not have any airway-related issues. Prolonged surgery in the steep Trendelenburg position can increase ICP and lead to post-extubation stridor, delayed awakening, visual loss, nerve injuries, and the migration of the endotracheal tube [6,7]. Post-reperfusion release of pneumoperitoneum, the reversal of the steep Trendelenburg position, and the use of mannitol and frusemide probably ensured the normalization of ICP and helped in “on-table extubation” in 92% of patients.

Conversion to open occurred in 3% of cases. It was essential in these cases to undock the robot emergently. Our team's familiarity with the emergency undocking helped us tide over this crisis. The mortality rate (3%) and graft survival (93%) in our series were comparable to previously published data in the literature [8].

DGF is defined as an acute renal failure of transplanted kidneys due to ischemic injury exacerbated by the reperfusion syndrome, which leads to oliguria, acute rejection, enhanced graft immunogenicity, and increased ICU and hospital stay [3]. The incidence of DGF in RAKT was found to be 3.6% by Oberholzer et al. in a cohort of 39 obese patients [9]. Musquera et al., in their multicenter prospective observational study of RAKT involving 291 patients, found the incidence of DGF to be 2% [10]. Incidence rates of 3.3% and 9.1% were reported in various other studies [11,12]. Prolonged CIT, rewarming time, surgical time, and console time can be risk factors for RAKT [10]. Seven (7%) patients had DGF in our series.

Cadaveric transplants have been associated with a higher incidence of DGF due to a higher incidence of donor hemodynamic fluctuations and prolonged CIT [3]. We did not find any association between the type of graft and DGF, probably due to the small number of deceased donors in our series. Although the creatinine levels were higher in deceased donors on days three, five, seven, and at one month, it was comparable at the end of one year.

The addition of epidural to GA produced a threefold increase in DGF rates [13]. This was attributed to epidural-induced vasodilatation and reduction in catecholamines reducing graft perfusion. Our method of GA with postoperative fentanyl infusion for pain relief may have proven to be a better technique as far as DGF is concerned.

Ojo et al. [13] studied 37,216 cadaveric renal transplants and concluded that every six hours of CIT increased the risk of DGF by 23%. The longer the CIT, the greater the chances of ischemia-reperfusion injury (IRI) with increased release of cytokines and free radicals. RAKT has been associated with longer CIT. However, similar to the other studies, we could not identify CIT as a risk factor for DGF (p=0.935) [10].

Liu et al., in their meta-analysis of six nonrandomized studies, found that although the RAKT group had significantly higher rewarming time and total ischemia time, the incidence of DGF was comparable with open transplants [14]. Although the safe upper limit of WIT is not known, prolonged WIT has been associated with increased DGF. Szostek et al. concluded that kidney temperature greater than 15 °C is a risk factor for DGF [15]. Gavela Martínez et al. retrospectively studied patients who developed DGF and found prolonged anastomosis time to be a risk factor [16].

The application of intraoperative regional hypothermia has shown a clear benefit when the anastomosis takes more than 40 minutes. The use of ice slush around the graft in our cohort helped in maintaining graft temperature. The WIT (p=0.135) and rewarming time (p=0.295) did not show any statistically significant difference between the two groups. Our findings corroborated that neither surgical time nor rewarming time showed any correlation with postoperative serum creatinine levels in RAKT [10].

A restrictive fluid regimen produced a fourfold increase in the risk of DGF. Liberal fluid therapy before reperfusion resulted in higher blood pressures, early diuresis, and early graft function [3,13]. A major constraint in RAKT is the absence of a reliable index of fluid responsiveness. CVP has been used as a standard for optimizing fluid therapy in open renal transplants [17]. The intraoperative increases in CVP (14 ± 4 mmHg), pulmonary arterial pressure (PAP), and pulmonary capillary wedge pressures (PCWP) are due to pneumoperitoneum and head-tilt rather than changes in the intrathoracic blood volume [2]. Stroke volume variability (SVV), pulse pressure variability (PPV), pleth variability index (PVI), and PCWP reflected fluid responsiveness poorly in laparoscopic surgeries [18-20].

Hence, we developed an institutional fluid management protocol. We did not find any association between the volume or type of crystalloids used and DGF. We also used frusemide (100 mg) to induce diuresis. Mannitol (0.25-0.5 g/kg) was used prior to reperfusion for its volume-expanding and osmotic diuretic effects. Of note, 93% of patients had immediate adequate UOP in our cohort. The estimated glomerular filtration rate (GFR) at one month in our RAKT series (64.4 mL/min/1.73 m^2^) was comparable with our open transplant patients (68.37 mL/min/1.73 m^2^) where the fluid management was CVP-based. The total fluids used or the type of fluids did not have an influence on DGF in our cohort.

We found a correlation between DGF and recipient age, dyslipidemia, and diabetes mellitus. Lebranchu et al. found that the recipient age >55 years increases the risk of DGF [21]. The average age of recipients with DGF in our cohort was 50.4 ± 6.6 years, which was less than what was reported in the literature. The average duration of ICU and hospital stay was significantly higher in patients with DGF in our cohort, in line with findings in the published data [22].

Similar to our findings, diabetes and dyslipidemia have been upheld as risk factors for DGF in other studies as well [23-25]. Damodaran et al. found that the chance of developing DGF increases by 50% when the recipient has diabetes [25]. This is particularly significant because diabetes is one of the leading causes of end-stage renal disease [23]. Various theories have been proposed to explain the mechanism by which diabetes increases DGF. Diabetes predisposes to atherosclerosis and hence increases the technical difficulty during surgery and increases the ischemia time. Increased susceptibility of diabetic patients to cardiovascular events, hemodynamic instability, chronic inflammation, and oxidative stress associated with diabetes can augment ischemia-reperfusion injury and increase the chances of DGF. Hyperglycemia without diabetes has also been reported to increase the chances of DGF [24,25]. Appropriate immunosuppression, control of blood sugars, and maintenance of hemodynamic stability should receive greater emphasis in diabetic patients.

Pretransplant lipoprotein disturbances have been found to influence early graft function [25]. Poor quality of vessels along with ongoing inflammation can affect the anastomosis time, thereby increasing the risk for DGF. Similar to diabetes, dyslipidemia can increase cardiovascular instability.

We compared the duration of anesthesia, surgery, and console time between the two groups and did not find any statistically significant association. Pneumoperitoneum can reduce GFR and urine output is reduced due to renal ischemia [ 4]. However, Parikh et al., in their study on laparoscopic renal transplants, did not find any adverse effects of pneumoperitoneum on renal allograft [2]. Animal studies have also shown that the effect of pneumoperitoneum is less on hydrated donor kidneys [10]. Post-reperfusion, the modification of the surgical technique to completely release the pneumoperitoneum ensured adequate blood flow to the newly grafted kidney. This obliterated the potential negative effect of high intra-abdominal pressure on the neo-renal microcirculation.

Despite the challenges we described, there are a number of advantages of RAKT. It is associated with smaller incisions and lower blood loss, tissue trauma, pain, and wound infection [9]. Earlier mobilization, better cosmesis, and early discharge can be added to the list of advantages of robotic-assisted surgeries. Fentanyl infusion of 0.5 mcg/kg/hour with intravenous paracetamol ensured adequate pain relief in all patients. None of our patients required rescue analgesics. All patients were mobile on the first postoperative day. The absence of wound infections in our series was remarkable in RAKT as these patients were immunosuppressed.

Our study has a few limitations. Firstly, it was retrospective in design, which could have led to some risk of detection bias and underreporting. Some unmeasured variables may have affected the accuracy of our model. Logistic regression model for postop dialysis with independent risk factors could not be used. Still, this study has important clinical implications as it was one of the first few to look into DGF in RAKT in the context of anesthesia.

## Conclusions

The incidence of DGF in our series was 7%, which is comparable with the available literature. Advanced recipient age, presence of diabetes, and dyslipidemia were factors associated with DGF in RAKT. However, CIT, rewarming time, console time, duration of surgery and Trendelenburg position, intraoperative fluid therapy, and type of graft did not have any association with DGF in our study. A thorough preoperative evaluation and optimization, knowledge and anticipation of potential problems and meticulous intraoperative fluid management, appropriate monitoring, and a multispecialty team approach are essential for the efficient anesthetic management of these complex procedures.
